# Beyond Conventional Analgesics: Updated Evidence Supporting Botulinum Toxin Type A for-Neuralgia Management

**DOI:** 10.7150/ijms.130934

**Published:** 2026-03-17

**Authors:** Jui-Ting Yu, Chen-Pi Li, Tsai-Mei Huang, Ru-Yin Tsai

**Affiliations:** 1 Division of Hematology/Medical Oncology, Department of Medicine, Tungs' Taichung MetroHarbor Hospital, Taichung 43503, Taiwan.; 2 Department of Nursing, Tungs' Taichung MetroHarbor Hospital, Taichung 43503, Taiwan.; 3 Department of Nursing, Hungkuang University, Taichung 43304, Taiwan.; 4 Department of Anatomy, School of Medicine, Chung Shan Medical University, Taichung 40201, Taiwan.; 5 Department of Medical Education, Chung Shan Medical University Hospital, Taichung 40201, Taiwan.

**Keywords:** neuropathic pain, pain intensity, sleep disturbance, inflammatory.

## Abstract

**Background:**

Neuralgia is a chronic neuropathic pain condition characterized by recurrent paroxysms of severe pain that are often refractory to conventional pharmacological therapies. Botulinum toxin type A (BTX-A) has emerged as a promising adjunctive treatment due to its analgesic and neuromodulatory properties.

**Objective:**

Evidence from randomized controlled trials (RCTs) remains heterogeneous, with variability in dosage, injection protocols, and follow-up duration. In addition, prior meta-analyses lacked detailed subgroup analyses, and several RCTs combined BTX-A with concomitant long-term analgesics, obscuring its independent therapeutic effect. This study aimed to provide a comprehensive synthesis of the efficacy and safety of BTX-A monotherapy for neuralgia, while permitting the use of acute analgesics (PROSPERO registration: CRD420251042926).

**Methods:**

We systematically searched PubMed, Embase, the Cochrane Library, and Web of Science for RCTs published up to September 2025 that evaluated BTX-A monotherapy in adults with neuralgia. Study selection and data extraction adhered to PRISMA 2020 guidelines. Pooled effect sizes were calculated using random-effects models, and heterogeneity was quantified with the *I²* statistic. Prespecified subgroup analyses were conducted based on dosage and follow-up duration.

**Results:**

Seven RCTs encompassing 368 participants were included. Compared with placebo or standard therapy, BTX-A significantly reduced pain intensity (VAS), attack frequency, and rescue analgesic use, while improving sleep quality, quality of life (QoL), and patient global impression of change (PGIC). Subgroup analysis revealed that low-dose regimens (<50 U) achieved comparable analgesic efficacy and durability to higher doses, with effects sustained for approximately three months. No serious adverse events were reported.

**Conclusions:**

BTX-A monotherapy provides clinically meaningful pain relief and functional improvement in patients with neuralgia, including trigeminal and postherpetic neuralgia. Its favorable safety profile and sustained efficacy support its role as an effective adjunct or alternative for refractory neuropathic pain. Future large-scale RCTs with standardized dosing and extended follow-up are warranted to optimize treatment protocols.

## Introduction

Neuralgia represents a disabling form of neuropathic pain characterized by recurrent, often severe paroxysms that significantly impair daily functioning and quality of life. Among its subtypes, trigeminal neuralgia (TN) and postherpetic neuralgia (PHN) are two of the most prevalent and debilitating conditions 1. Despite the availability of pharmacological treatments such as carbamazepine, gabapentin, and pregabalin, many patients experience incomplete relief or intolerable adverse effects, highlighting the urgent need for novel therapeutic strategies that can provide sustained analgesia with favorable tolerability profiles 2, 3.

Botulinum toxin type A (BTX-A), a neurotoxin produced by Clostridium botulinum, has emerged as a promising therapeutic agent beyond its traditional role in treating dystonia and muscle spasticity 4. Its analgesic effects are thought to be mediated by inhibition of neurotransmitter release, including substance P, calcitonin gene-related peptide (CGRP), and glutamate, thereby modulating both peripheral and central sensitization processes 5. Experimental studies and clinical observations have demonstrated that BTX-A can alleviate neuropathic pain through mechanisms involving the suppression of neurogenic inflammation and downregulation of transient receptor potential (TRP) channels 6, 7. Clinical trials have shown encouraging results in TN and PHN, where subcutaneous or intradermal injections of BTX-A provided significant pain reduction lasting several weeks to months 8-10.

However, inconsistencies remain among randomized controlled trials (RCTs) regarding dosing regimens, injection techniques, and follow-up durations 10-12. Furthermore, some studies combined BTX-A with conventional analgesics, complicating the assessment of its independent efficacy 3, 13. Previous meta-analyses have provided supportive but limited conclusions due to small sample sizes and lack of subgroup analyses exploring dose-response relationships or duration of benefit 3, 14, 15.

To address these gaps, the present study provides an updated systematic review and meta-analysis focusing exclusively on BTX-A monotherapy for the treatment of neuralgia, encompassing both TN and PHN. This comprehensive synthesis aims to clarify the magnitude and durability of BTX-A's analgesic effects, evaluate optimal dosing strategies, and assess its safety profile.

## Materials and Methods

### Data sources and selection criteria

A comprehensive literature search was conducted in PubMed, Embase, the Cochrane Library, and Web of Science to identify eligible RCTs published up to September 2025. The search strategy combined Medical Subject Headings (MeSH) and free-text terms related to “botulinum toxin type A”, “neuralgia”, “neuropathic pain”, “trigeminal neuralgia”, and “postherpetic neuralgia”. Reference lists of relevant reviews and included studies were manually screened to ensure completeness.

### Selection of studies

**Figure 1:**
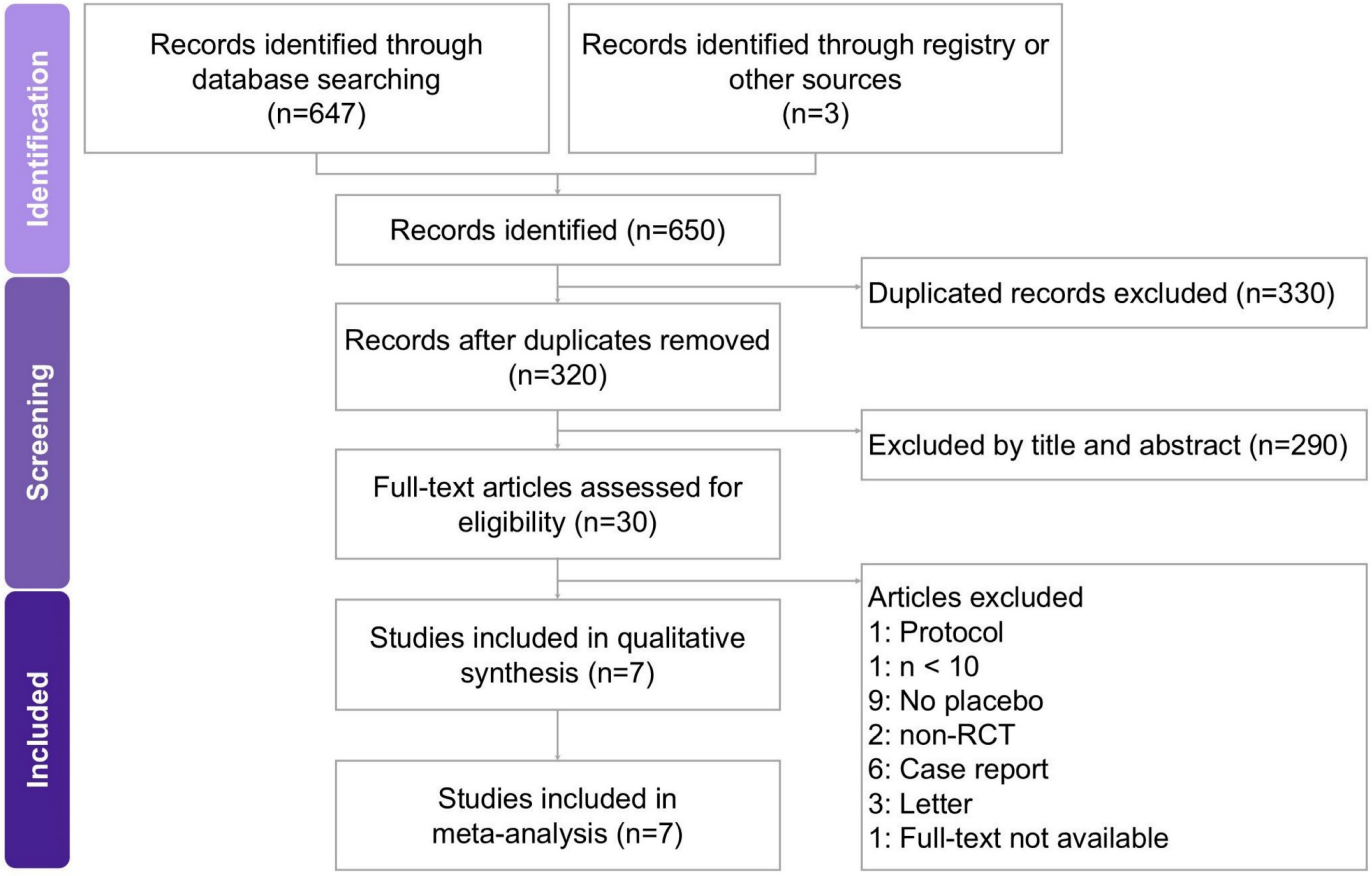
** A flowchart illustrating the study selection process for the systematic review and meta-analysis on the effects of BTX-A intervention in managing neuralgia.** Of the 650 records initially identified, 7 studies met the eligibility criteria and were included in the final analysis.

Two researchers independently conducted the screening and assessment of eligible studies, with a third researcher providing oversight to maintain accuracy and consistency throughout the process. All relevant articles were obtained in full text and carefully examined to ensure thorough evaluation. The step-by-step selection procedure is illustrated in the PRISMA flow diagram (Figure 1). Studies were included if they met the following criteria: (1) randomized controlled trials evaluating botulinum toxin type A monotherapy for neuralgia in adult participants; (2) diagnosis of trigeminal neuralgia or postherpetic neuralgia established by recognized clinical criteria; (3) reported at least one quantitative outcome measure, such as pain intensity (VAS), pain frequency, analgesic consumption, or patient-reported outcomes (sleep quality, QoL, or PGIC); and (4) comparator groups receiving placebo or standard therapy without BTX-A.

### Data extraction

Two researchers independently extracted data using a standardized form based on the Cochrane Handbook recommendations 16. Exclusion criteria included: (1) non-RCT or uncontrolled designs; (2) studies combining BTX-A with other active interventions (e.g., gabapentin, carbamazepine) in both groups without isolating BTX-A effects; (3) insufficient outcome data; and (4) duplicate publications, abstracts, reviews, case reports, and animal studies.

### Outcomes

The primary outcomes assessed in this study included pain intensity, measured by the VAS, and pain attack frequency. Secondary outcomes encompassed rescue analgesic use, sleep quality, QoL, and PGIC. Additional safety outcomes, including the incidence and severity of adverse events, were extracted when available. Prespecified subgroup analyses were conducted based on BTX-A dosage (low dose ≤50 U vs. high dose >50 U) and follow-up duration (8 weeks, 12 weeks, and 24 weeks) to explore potential sources of heterogeneity and to evaluate the sustainability of analgesic effects over time.

### Assessment of methodological quality

Risk of bias was independently assessed by two reviewers using the Cochrane Risk of Bias tool (RoB 2). Each study was evaluated across the predefined methodological domains recommended by the Cochrane Collaboration. Any disagreements were resolved through discussion, with consultation from a third reviewer when necessary. The assessment followed established RoB 2 guidance to ensure consistency and methodological rigor.

### Data analysis

Quantitative synthesis was performed by calculating standardized mean differences (SMDs) with corresponding 95% confidence intervals (CIs) to compare continuous outcomes between the intervention and control groups. A random effects model was used to account for potential clinical and methodological variability among studies. All statistical analyses were conducted using Comprehensive Meta-Analysis (CMA), version 3 (Biostat, Englewood, NJ, USA). Heterogeneity across studies was quantified using the* I²* statistic, with values greater than 50% considered indicative of substantial heterogeneity. Publication bias was evaluated through funnel plot inspection and Egger's regression test, with a significance threshold of *p* < 0.05 for most outcomes and *p* < 0.10 for bias detection. Subgroup analyses were carried out to explore potential sources of heterogeneity, and sensitivity analyses were conducted by sequentially excluding individual studies to assess the robustness of the pooled estimates.

## Results

### Study search and characteristics of included patients

The study selection process is illustrated in Figure 1. A comprehensive search of PubMed, Embase, the Cochrane Library, and Web of Science initially yielded 650 potentially relevant records. After removing duplicates, 320 unique articles were screened based on titles and abstracts, of which 290 were excluded for not meeting the inclusion criteria. The remaining 30 full-text articles were reviewed in detail, resulting in the exclusion of 23 studies for the following reasons: unrelated protocols 17, sample size fewer than 10 participants 18, non-randomized design 10, 19, case reports 20-25, letters or correspondence 26-28, full-text not available 18, and lack of a placebo or control group 8, 29-36. Ultimately, seven RCTs published between 2010 and 2022 were included in this meta-analysis, encompassing a total of 368 participants. All eligible 9-12, 37-39 studies evaluated the therapeutic efficacy of BTX-A for the treatment of neuralgia, specifically including cases of trigeminal neuralgia and postherpetic neuralgia. The principal characteristics of the included trials and patient demographics are summarized in Table 1.

### Quality assessment

**Figure 2:**
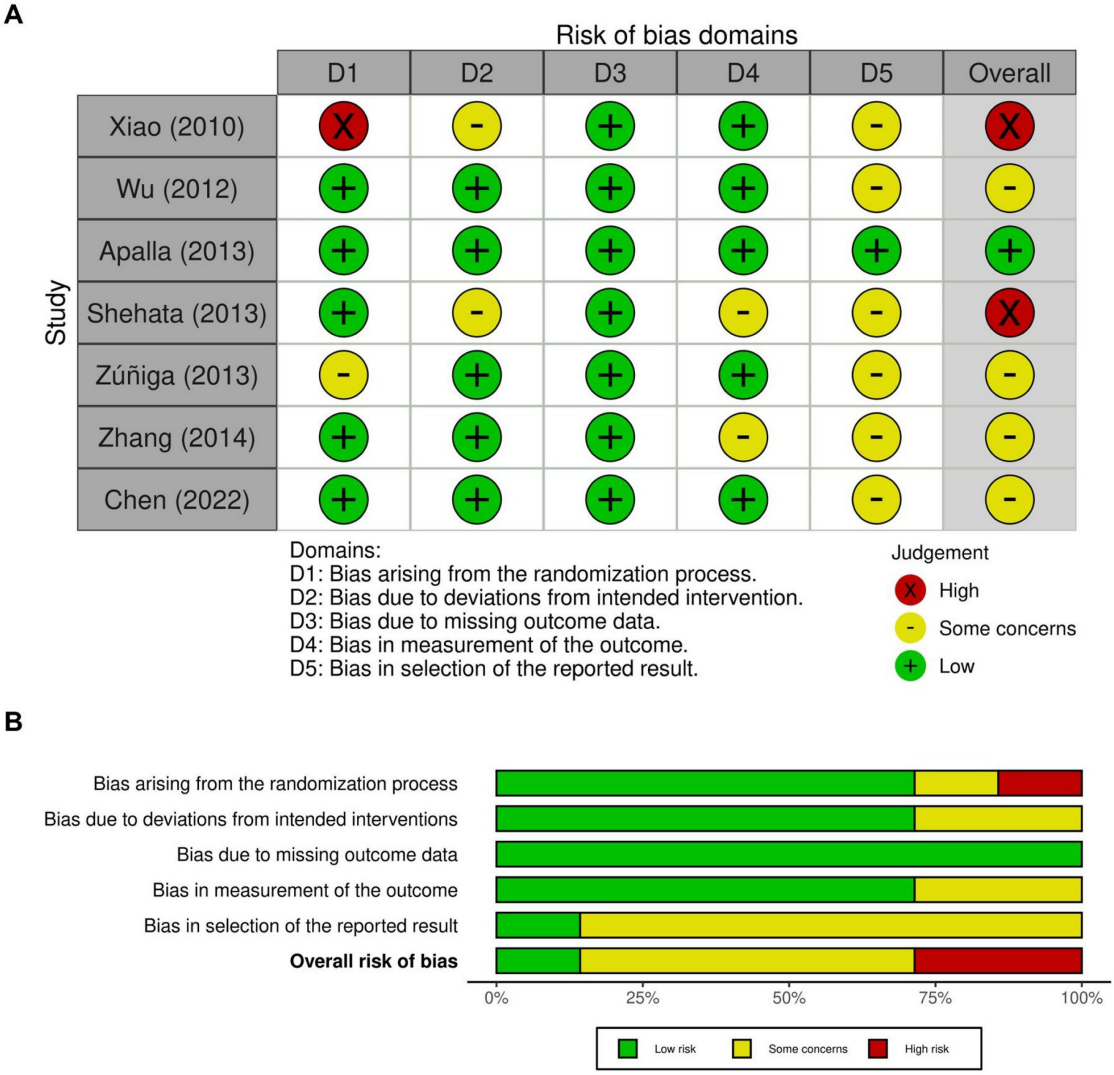
** Evaluation of the methodological quality of the included trials.** (A) Individual risk of bias assessment for each selected study, based on the Rob 2.0 tool (https://mcguinlu.shinyapps.io/robvis/). (B) The overall risk of bias was quantified as a percentage. The most frequent sources of high risk among the included studies were deviations from the randomization process and concerns regarding the selection of reported outcomes.

The methodological quality of the seven included RCTs was evaluated using the Cochrane Collaboration's Risk of Bias 2 (RoB 2) tool. Overall, the studies demonstrated acceptable methodological rigor with clearly defined eligibility criteria, appropriate randomization methods, and comparable baseline characteristics across treatment groups. Most trials exhibited a low risk of bias in domains related to randomization, missing outcome data, and outcome measurement. However, several studies presented some concerns regarding deviations from intended interventions, primarily due to challenges in maintaining complete blinding during injections. In certain trials, the presence of visible local effects or differences in injection sensations may have partially compromised participant blinding, introducing potential performance bias. A few studies, such as those by Xiao 9 and Shehata 37, showed a high risk of bias within specific domains, particularly concerning randomization procedures or unclear allocation concealment. Nevertheless, outcome measurements were generally based on standardized clinical scales (VAS or PGIC) and were assessed by trained evaluators, minimizing detection bias. Reporting bias was judged to be low in nearly all studies, as pre-specified outcomes were consistently reported. The overall assessment indicated that most trials were of moderate to high methodological quality, with minimal risk identified in randomization and data integrity. The distribution of bias across domains is illustrated in Figure 2, where Figure 2A depicts the domain-level judgments for each study, and Figure 2B summarizes the proportion of studies rated as low risk, some concerns, or high risk.

### Effect of BTX-A on VAS

**Figure 3:**
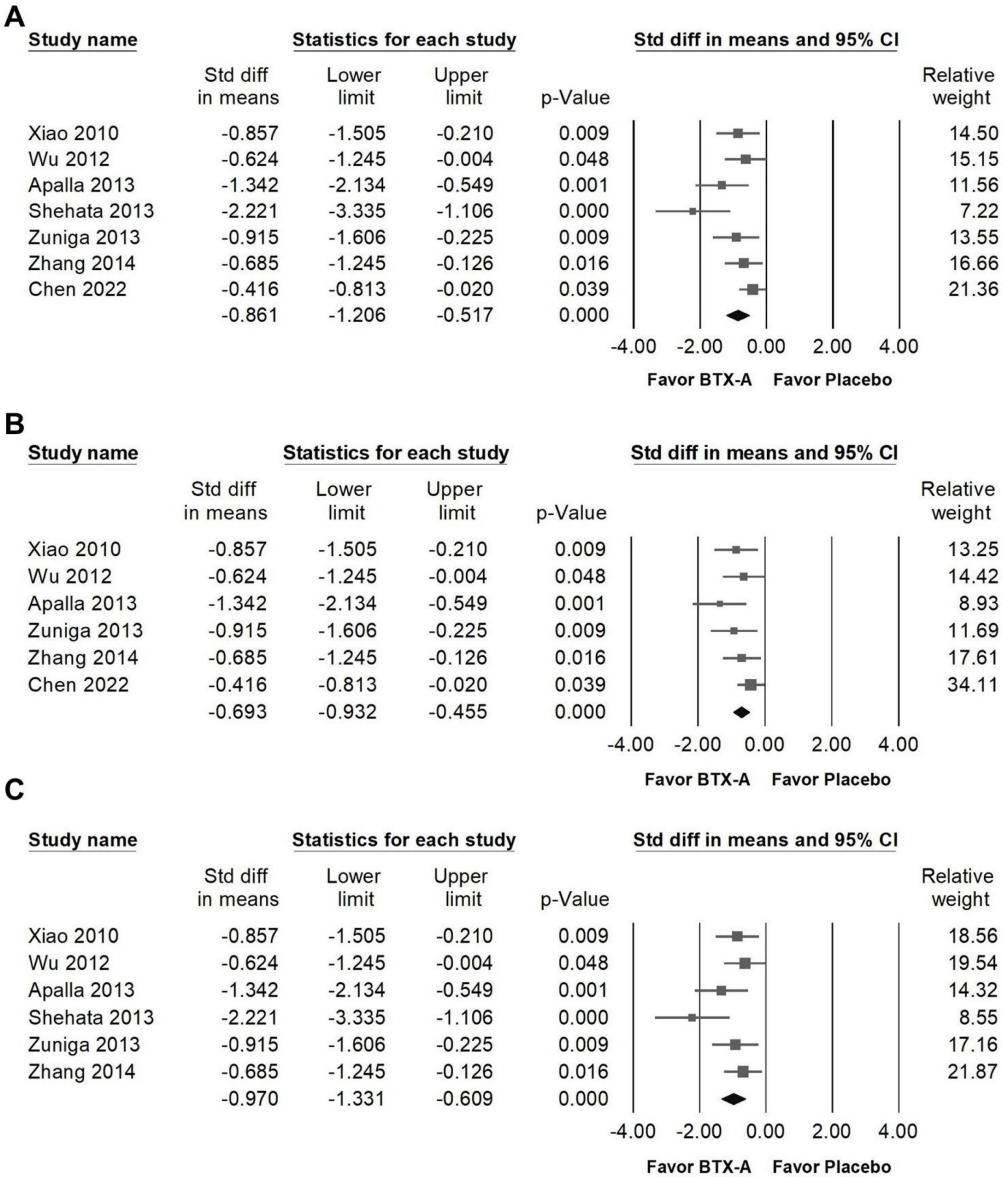
** Forest plots illustrating the effects of BTX-A intervention on pain intensity measured by the Visual Analog Scale (VAS).** Panel (A) presents the pooled effects across all included studies, whereas panels (B) and (C) display the results of sensitivity analyses conducted after excluding the outlier studies by Shehata (2013) and Chen (2022), respectively.

BTX-A supplementation was associated with a great reduction in pain intensity measured by VAS (Figure 3A, SMD = -0.861, 95% CI: -1.206 to -0.517, *p* < 0.001; *I²* = 50.377%, *p* = 0.060). Among the included studies, Shehata et al. 37 demonstrated an effect size that differed substantially from the other RCTs. To assess the influence of this study, a sensitivity analysis was performed after its exclusion. The pooled estimate remained statistically significant, indicating a moderate reduction in VAS scores (Figure 3B, SMD = -0.693, 95% CI: -0.932 to -0.455, *p* < 0.001; *I²* =2.733%, *p* = 0.399). In contrast, Chen et al. 11 showed the smallest treatment effect among the included trials. Therefore, an additional sensitivity analysis was conducted excluding this study. The pooled result remained statistically significant and demonstrated a great reduction in VAS (Figure 3C, SMD = -0.970, 95% CI: -1.331 to -0.609, *p* < 0.001; *I²* = 36.746%, *p* = 0.162), further supporting the robustness of the overall findings.

**Figure 4:**
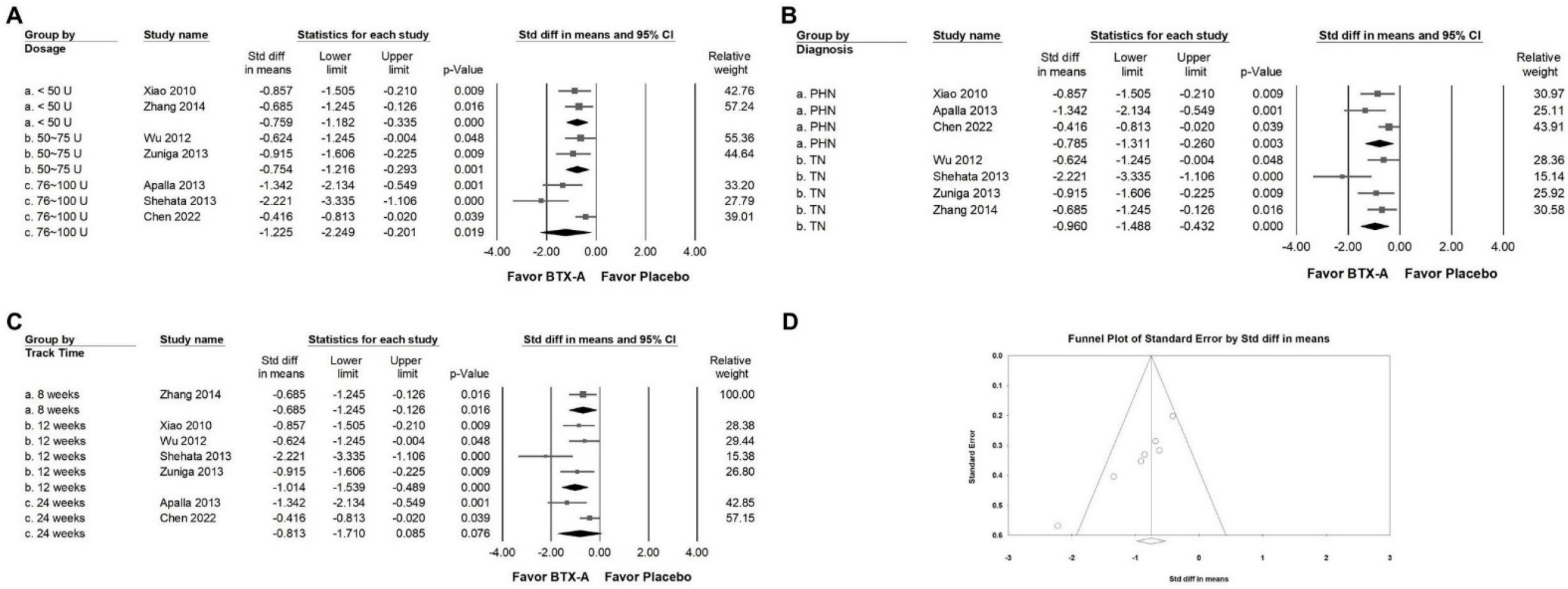
** Subgroup analyses corresponding to the effects presented in Figure 3A.** Panel (A) compares the effects according to the dosage of BTX-A intervention, panel (B) illustrates subgroup results by diagnosis, and panel (C) presents analyses stratified by follow-up duration. Each square represents an individual study, with its position indicating the standardized mean difference; squares positioned to the left indicate a reduction in pain intensity. Horizontal lines denote the 95% confidence intervals, and the diamonds at the bottom represent the pooled effect size for each subgroup analysis. Panel (D) presents a funnel plot evaluating potential publication bias among the studies included in Figure 3A. Each circle represents an individual study, with the size of the circle corresponding to its relative weight or sample size. The diagonal lines indicate the 95% confidence limits around the pooled effect estimate, while the central diamond denotes the overall summary effect and its confidence interval.

In the subgroup analysis stratified by intervention dosage (Figure 4A), BTX-A supplementation demonstrated a moderate reduction in VAS scores in studies using doses below 50 U (SMD = -0.759, 95% CI: -1.182 to -0.335, *p* < 0.001; *I²* < 0.001%, *p* = 0.694) and in those using 50 to 75 U (SMD = -0.754, 95% CI: -1.216 to -0.293, *p* = 0.001; *I²* < 0.001%, *p* = 0.539). Although a great effect size was observed in studies administering 76 to 100 U (SMD = -1.225, 95% CI: -2.249 to -0.201, *p* = 0.019; *I²* = 86.696%, *p* = 0.003), there was substantial heterogeneity among these studies, indicating variability in treatment response and study design. In the subgroup analysis stratified by diagnosis (Figure 4B), BTX-A demonstrated differential efficacy across neuralgia subtypes. In studies enrolling patients with PHN, BTX-A produced a moderate reduction in pain intensity (SMD = -0.785, 95% CI: -1.311 to -0.260, *p* = 0.003; *I²* = 57.092%, *p* = 0.098). In contrast, TN reported a greater analgesic effect (SMD = -0.960, 95% CI: -1.488 to -0.432, *p* < 0.001; *I²* = 55.086%, *p* = 0.083). These results suggest that BTX-A may provide stronger pain relief in TN than in PHN, although both subgroups demonstrated clinically meaningful improvement. In subgroup analyses based on follow-up duration (Figure 4C), BTX-A exhibited consistent analgesic efficacy across different observation periods. Studies with 8-week follow-up demonstrated a moderate reduction in pain intensity (SMD = -0.685, 95% CI: -1.245 to -0.126, *p* = 0.016; *I²* < 0.001%, *p* > 0.999). Similarly, trials with 12-week follow-up showed a significant decrease in VAS scores (SMD = -1.014, 95% CI: -1.539 to -0.489, *p* < 0.001; *I²* = 51.102%, *p* = 0.105). In contrast, studies with extended follow-up beyond 24 weeks did not demonstrate statistically significant improvement (SMD = -0.813, 95% CI: -1.710 to 0.085, *p* = 0.076; *I²* = 76.147%, *p* = 0.041). Based on the currently available RCTs, the analgesic effects of BTX-A appear to be most pronounced within the first three months following injection. However, only two trials have evaluated outcomes beyond 12 to 16 weeks. Therefore, the long-term efficacy of repeated or sustained BTX-A administration should be interpreted with caution and requires further investigation through adequately powered studies.

### Publishing bias

Egger's regression analysis suggested the presence of small-study effects (p = 0.00069). Visual inspection of the funnel plot for VAS (Figure 4D) revealed asymmetry, indicating the potential for publication bias or other sources of small-study variability. To further explore this issue, a trim-and-fill analysis was conducted. The adjusted pooled effect size (SMD = -0.861; 95% CI: -1.201 to -0.517) remained comparable to the original estimate, with no meaningful change in effect direction or magnitude. Although statistical evidence of asymmetry was observed, the consistency of the pooled estimate after adjustment suggests that the overall findings are unlikely to be substantially altered by potential publication bias. Nonetheless, these results should be interpreted with appropriate caution.

### BTX-A supplementation reduced pain frequency

**Figure 5:**
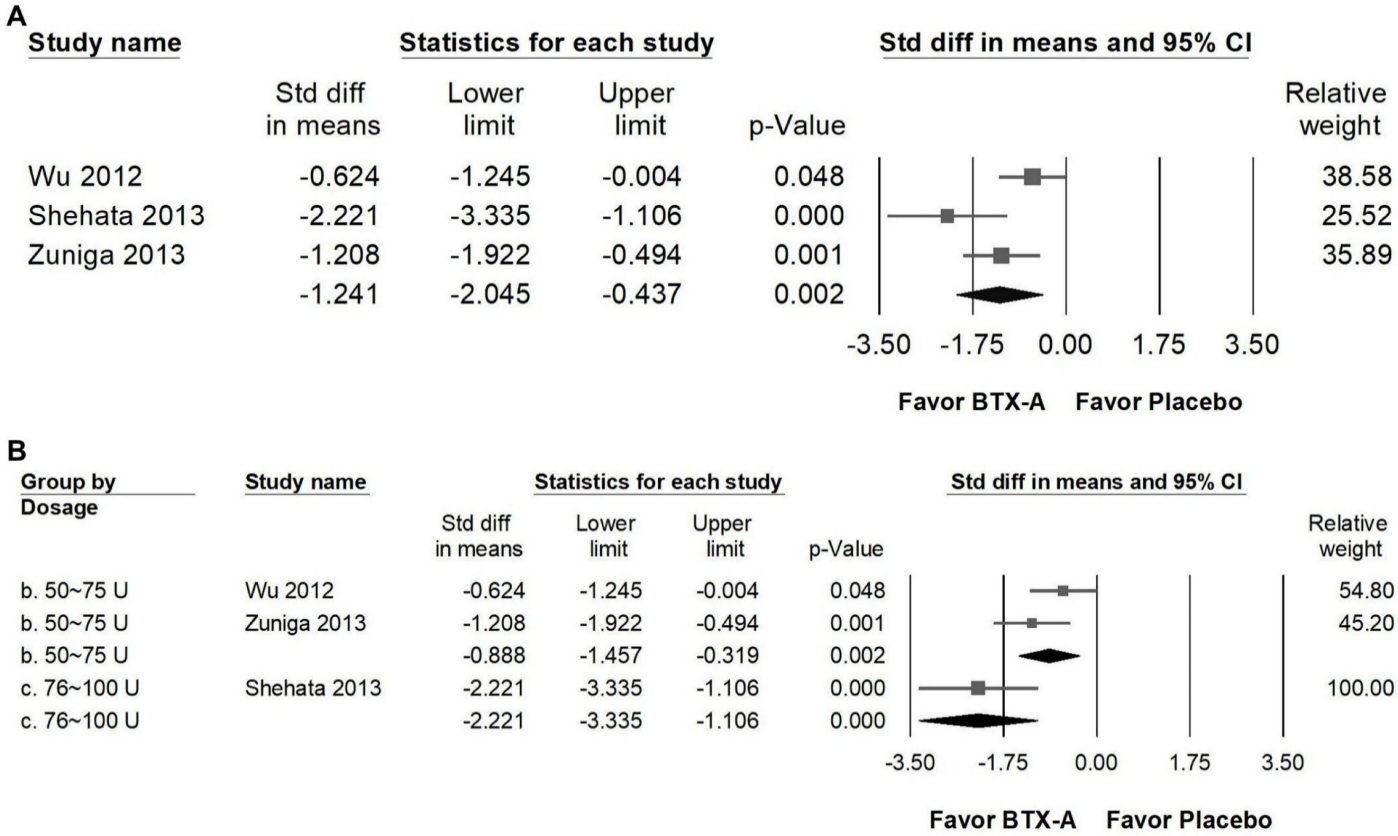
** Forest plots illustrating the effects of BTX-A intervention on pain frequency.** Panel (A) presents the pooled effects across all included studies, while panel (B) shows the subgroup analysis of panel (A) stratified by dosage. Each square represents the standardized mean difference of an individual study, with horizontal lines indicating the 95% confidence intervals. The diamonds at the bottom of each panel denote the pooled effect sizes, summarizing the overall effect of BTX-A intervention on pain frequency.

BTX-A supplementation produced a marked reduction in pain frequency compared with control groups (Figure 5A), showing a large effect size (SMD = -1.241, 95% CI: -2.045 to -0.437, *p* = 0.002; *I²* = 67.852%, *p* = 0.045). In subgroup analyses stratified by intervention dosage (Figure 5B), both 50 to 75 U and 76 to 100 U regimens demonstrated significant decreases in attack frequency. Studies using 50-75 U of BTX-A reported a great effect (SMD = -0.888, 95% CI: -1.457 to -0.319, *p* = 0.002; *I²* = 31.559%, *p* = 0.227), whereas the single trial using 76-100 U also showed a great reduction in pain frequency (SMD = -2.221, 95% CI: -3.355 to -1.106, *p* < 0.001; *I²* < 0.001%, *p* > 0.999). However, as this higher-dose subgroup was represented by only one trial, the findings should be considered exploratory and interpreted with caution. Overall, the available evidence suggests that BTX-A may reduce the recurrence of pain episodes; however, current data are insufficient to establish a definitive dose-response relationship.

### BTX-A supplementation reduced acute analgesic use

**Figure 6:**
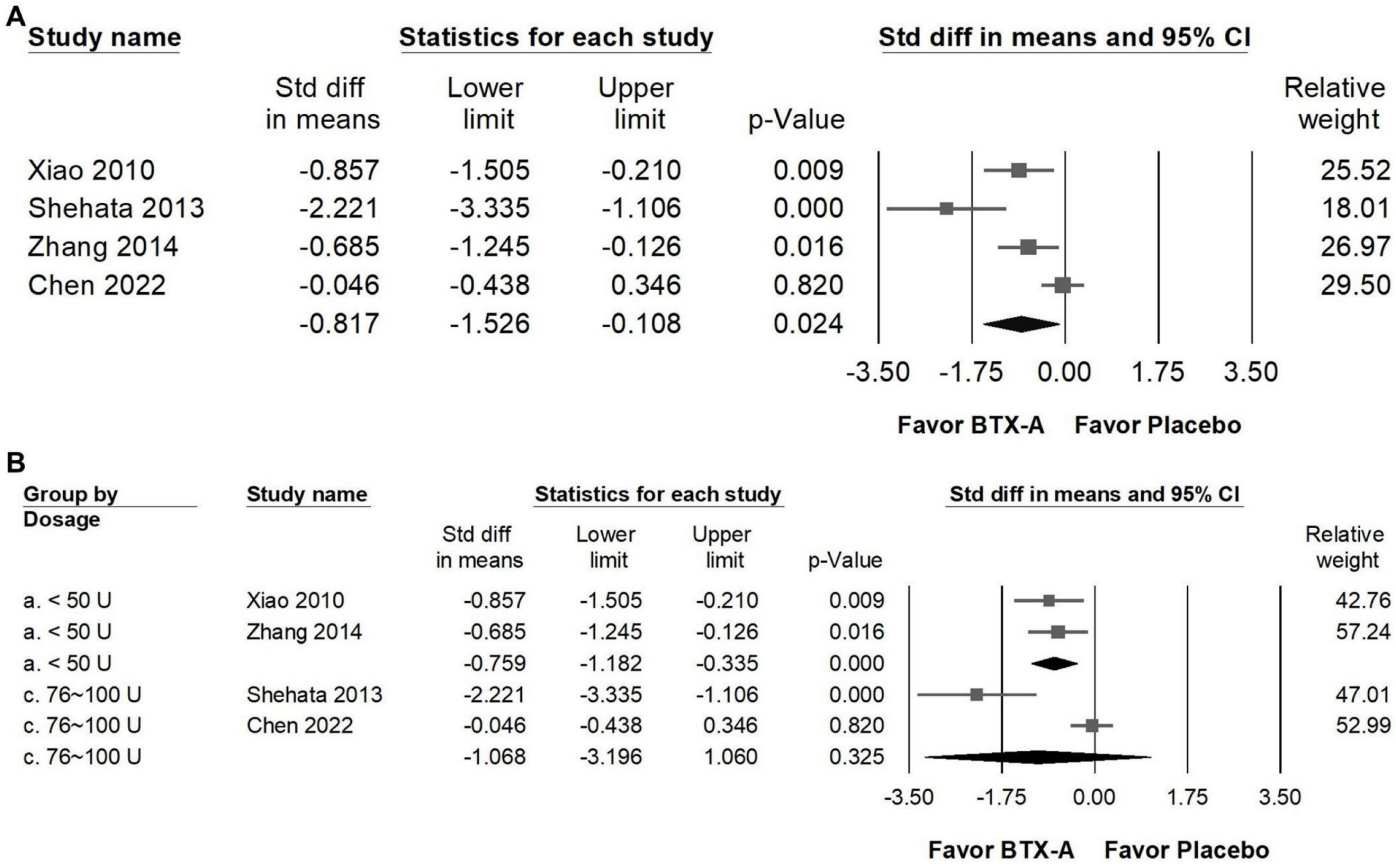
** Forest plots illustrating the effects of BTX-A intervention on the reduction of analgesic use.** Panel (A) presents the pooled effects across all included studies, while panel (B) displays the subgroup analysis of panel (A) stratified by dosage. Each square represents the standardized mean difference of an individual study, with horizontal lines indicating the 95% confidence intervals. The diamonds at the bottom of each panel denote the pooled effect sizes, summarizing the overall impact of BTX-A intervention on analgesic use reduction.

BTX-A supplementation significantly greatly reduced acute analgesic consumption compared with placebo, indicating a notable decrease in the need for rescue medications among patients receiving BTX-A treatment (Figure 6A). The pooled analysis revealed a great overall effect (SMD = -0.817, 95% CI: -1.526 to -0.108, *p* = 0.024) however, substantial heterogeneity was observed (*I²* = 81.138%, *p* = 0.001), indicating considerable variability across studies. In subgroup analyses stratified by dosage (Figure 6B), trials using less than 50 U of BTX-A showed a moderate reduction in analgesic use (SMD = -0.759, 95% CI: -1.182 to -0.335, *p* < 0.001; *I²* < 0.001%, *p* = 0.694), with minimal heterogeneity. In contrast, studies administering 76-100 U did not demonstrate a statistically significant difference compared with controls (SMD = -1.068, 95% CI: -3.196 to 1.060, *p* = 0.325), and heterogeneity in this subgroup was substantial (*I²* = 92.321%, *p* < 0.001). The high between-study variability may reflect differences in study design, baseline medication use, sample size, or patient characteristics. Given the limited number of studies and the presence of considerable heterogeneity, these findings should be interpreted cautiously, and no definitive dose-related conclusion can be drawn regarding analgesic consumption.

### Impact of BTX-A supplementation on sleep quality

**Figure 7:**
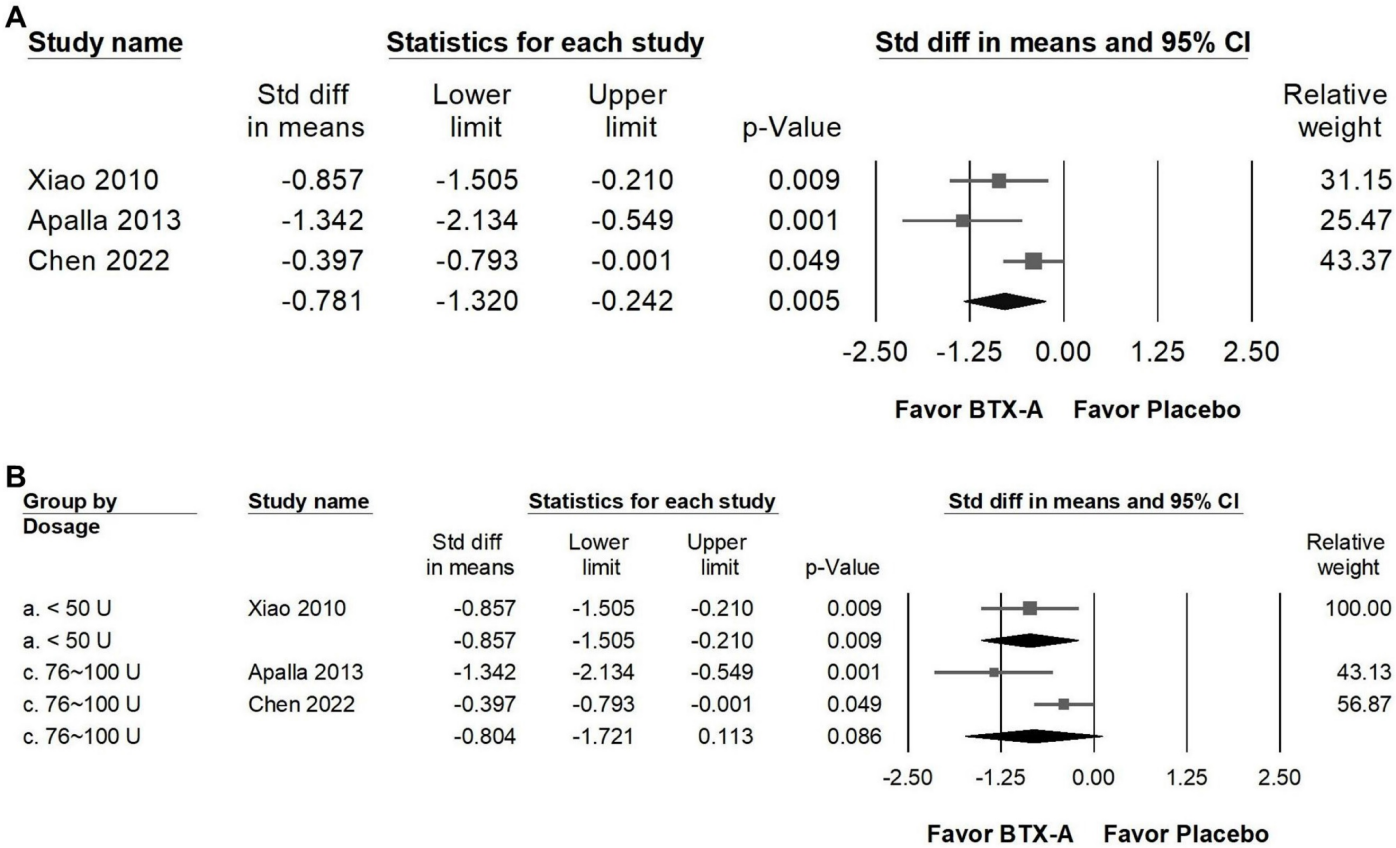
** Forest plots illustrating the effects of BTX-A intervention on sleep quality.** Panel (A) presents the pooled effects across all included studies, while panel (B) displays the subgroup analysis of panel (A) stratified by dosage. Each square represents the standardized mean difference of an individual study, with horizontal lines indicating the 95% confidence intervals. The diamonds at the bottom of each panel denote the pooled effect sizes, summarizing the overall impact of BTX-A intervention on sleep quality.

BTX-A supplementation was associated with a statistically significant improvement in sleep quality compared with control groups (Figure 7A). The pooled analysis demonstrated a moderate effect size (SMD = -0.781, 95% CI: -1.320 to -0.242, *p* = 0.005), although moderate heterogeneity was observed (*I²* = 59.122%, *p* = 0.087). This finding suggests a potential association between BTX-A treatment and improved sleep outcomes. In the dose-stratified subgroup analysis (Figure 7B), trials using less than 50 U of BTX-A demonstrated a statistically significant improvement in sleep quality (SMD = -0.857, 95% CI: -1.505 to -0.210, *p* = 0.009; *I²* < 0.001%, *p* > 0.999), with minimal heterogeneity. In contrast, studies administering 76-100 U did not reach statistical significance (SMD = -0.804, 95% CI: -1.721 to 0.113, *p* = 0.086), and substantial heterogeneity was observed in this subgroup (*I²* = 77.125%, *p* = 0.037). Given the limited number of studies and variability across dosage categories, these findings should be interpreted cautiously. While BTX-A appears to be associated with improvements in sleep quality, the available data does not support definitive conclusions regarding dose-related differences.

### Impact of BTX-A supplementation on life quality

**Figure 8:**
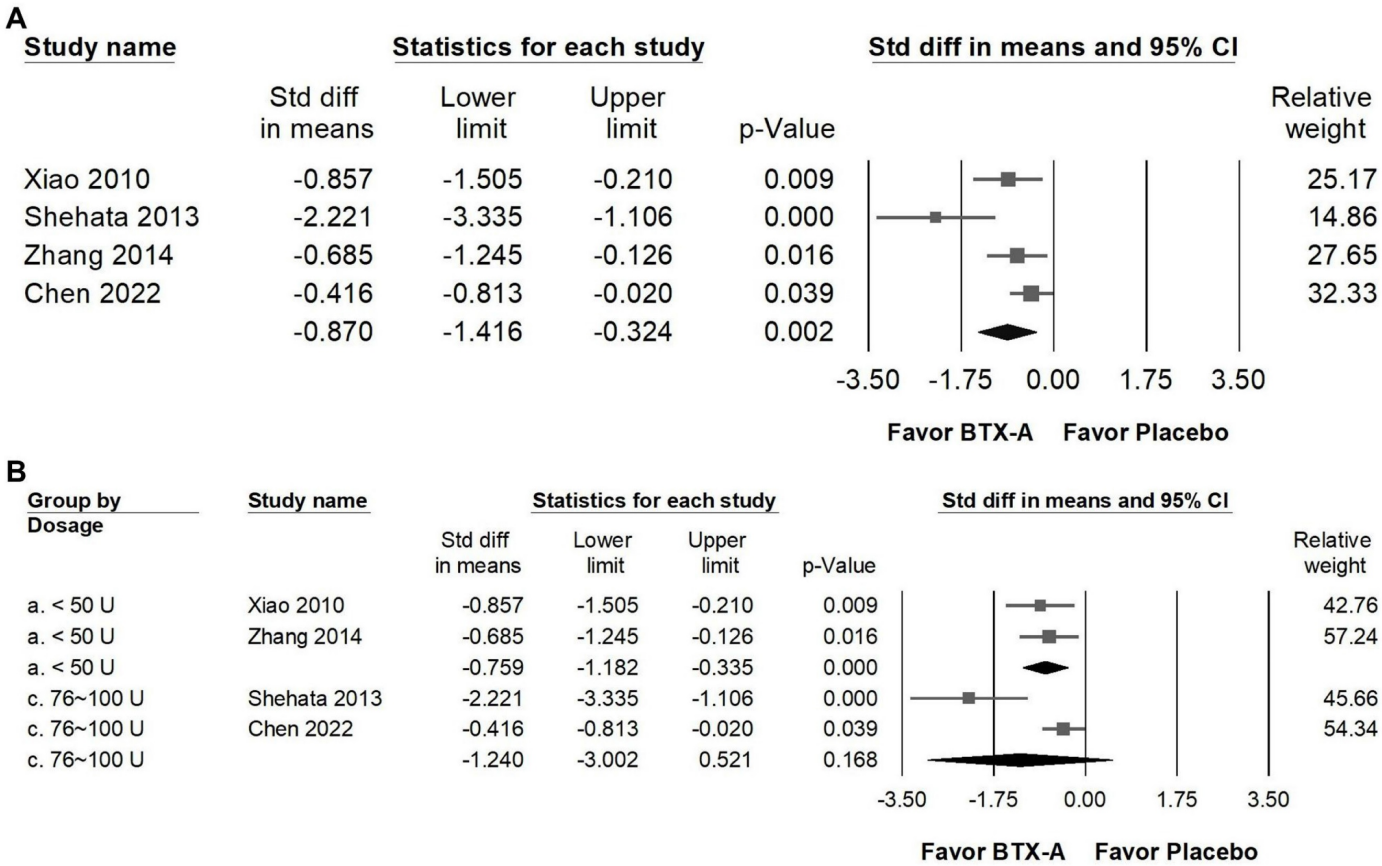
** Forest plots illustrating the effects of BTX-A intervention on quality of life (QoL).** Panel (a) presents the pooled effects across all included studies, while panel (b) displays the subgroup analysis of panel (a) stratified by dosage. Each square represents the standardized mean difference of an individual study, with horizontal lines indicating the 95% confidence intervals. The diamonds at the bottom of each panel denote the pooled effect sizes, summarizing the overall impact of BTX-A intervention on quality of life.

BTX-A supplementation was associated with a statistically significant improvement in sleep quality compared with control groups (Figure 8A; SMD = -0.870, 95% CI: -1.416 to -0.324,* p* = 0.002). However, substantial heterogeneity was observed (*I²* = 67.857%, *p* = 0.025), indicating variability across studies. In the dose-stratified subgroup analysis (Figure 8B), trials using less than 50 U of BTX-A demonstrated a statistically significant improvement (SMD = -0.759, 95% CI: -1.182 to -0.335, *p* < 0.001; *I²* < 0.001%, *p* = 0.694), with minimal heterogeneity. In contrast, studies administering 76-100 U did not show a statistically significant difference compared with controls (SMD = -1.240, 95% CI: -3.002 to 0.521, *p* = 0.168), and heterogeneity in this subgroup was considerable (*I²* = 88.816%, *p* = 0.003). Given the high between-study variability and the limited number of trials within the higher-dose subgroup, these findings should be interpreted cautiously, and no definitive conclusions can be drawn regarding dose-related differences in sleep outcomes.

### Impact of BTX-A supplementation on PGIC

**Figure 9:**
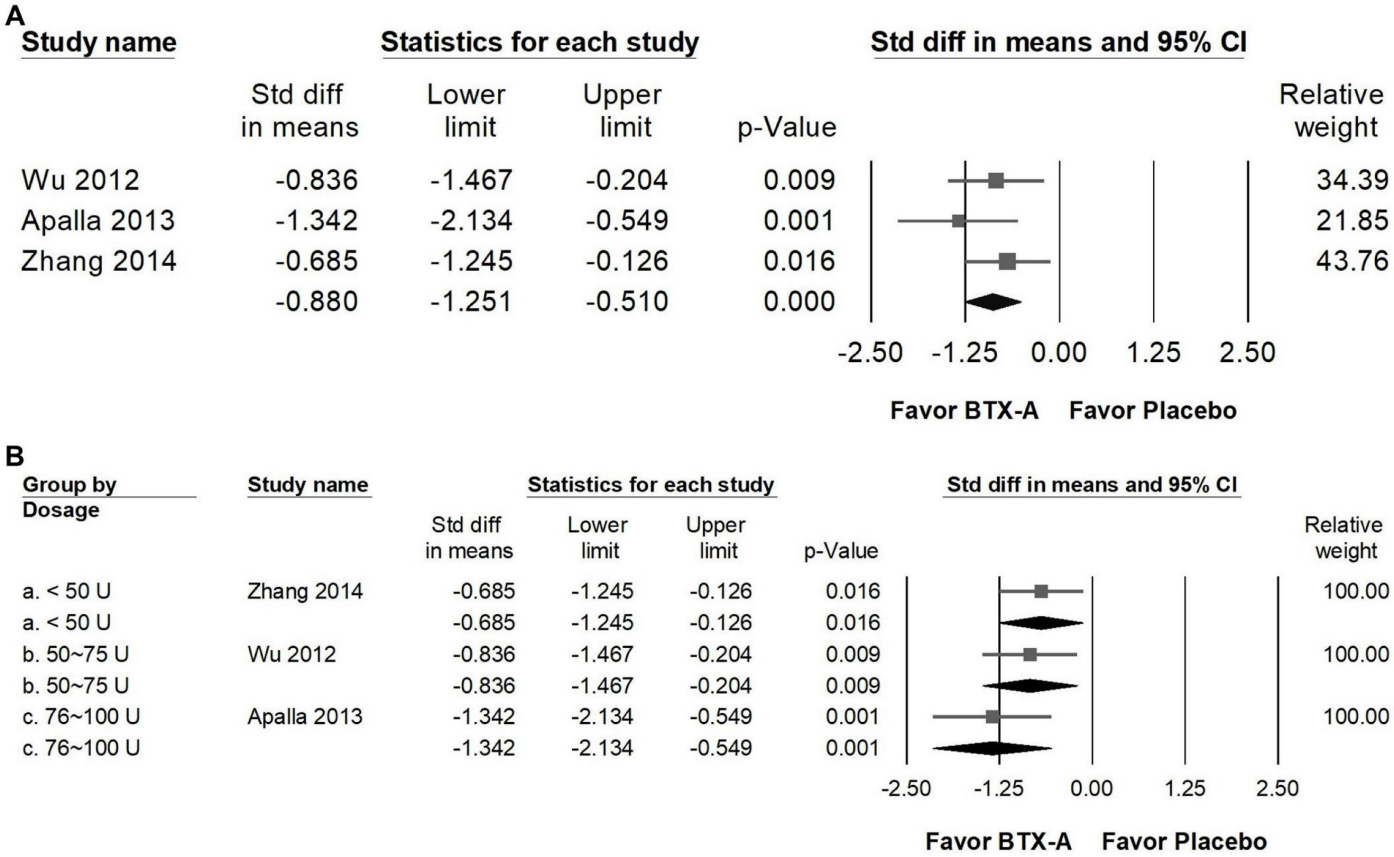
** Forest plots illustrating the effects of BTX-A intervention on Patient Global Impression of Change (PGIC).** Panel (a) presents the pooled effects across all included studies, while panel (b) displays the subgroup analysis of panel (a) stratified by dosage. Each square represents the standardized mean difference of an individual study, with horizontal lines indicating the 95% confidence intervals. The diamonds at the bottom of each panel denote the pooled effect sizes, summarizing the overall impact of BTX-A intervention on PGIC outcomes.

BTX-A supplementation resulted in a great improvement in patients' global impression of change (PGIC) compared with control groups (Figure 9A), reflecting a meaningful enhancement in patients' overall perception of symptom relief and treatment satisfaction. The pooled analysis showed a significant great effect (SMD = -0.880, 95% CI: -1.251 to -0.510, *p* < 0.001; *I²* < 0.001%, *p* = 0.409). In the subgroup analysis stratified by dosage (Figure 9B), studies using less than 50 U of BTX-A demonstrated a moderate effect (SMD = -0.685, 95% CI: -1.245 to -0.126, *p* = 0.016; *I²* < 0.001%, *p* > 0.999). In contrast, trials administering 50-75 U (SMD = -0.836, 95% CI: -1.467 to -0.204, *p* = 0.009; *I²* < 0.001%, *p* > 0.999) and 76-100 U (SMD = -1.342, 95% CI: -2.134 to -0.549, *p* = 0.001; *I²* < 0.001%, *p* > 0.999) both showed great effects in improving PGIC outcomes. However, it should be noted that each of these subgroups was represented by a single RCT, and thus the findings should be interpreted with caution due to limited data.

Our findings should be interpreted in the context of previous meta-analyses evaluating BTX-A in neuralgia. Earlier syntheses reported that BTX-A may provide benefit in trigeminal neuralgia and postherpetic neuralgia; however, they generally pooled heterogeneous treatment regimens and offered limited evaluation of dosage strata or follow-up duration 3, 15, 40.

For example, Shackleton 3 identified moderate evidence of efficacy in randomized placebo-controlled trials but did not examine the influence of dose or duration on outcomes. Subsequent reviews focusing on postherpetic neuralgia also supported benefit compared with active analgesics, yet combined diverse injection protocols and background therapies 15. A broader neuropathic pain meta-analysis similarly suggested therapeutic potential while emphasizing the need for standardized dosing strategies and longer follow-up 40. In the present analysis, subgroup exploration by dose and follow-up duration provides additional descriptive insight into patterns of response. Analgesic effects were observed across low and mid dose ranges, and several trials reported sustained benefits up to approximately twelve weeks. Nevertheless, given the limited number of trials within certain subgroups and the variability in study design, these observations should not be interpreted as establishing a definitive dose-response relationship or long-term durability profile.

Several mechanistic studies provide biological plausibility for the analgesic effects observed with BTX-A. Beyond inhibition of acetylcholine release at the neuromuscular junction, BTX-A has been shown to reduce the release of pain-related neurotransmitters such as substance P, glutamate, and calcitonin gene related peptide, thereby attenuating peripheral sensitization and neurogenic inflammation 5. Experimental data also suggest modulation of transient receptor potential channels and voltage-gated sodium channels, which are implicated in ectopic neuronal firing within the trigeminal system 41. These molecular effects may contribute to clinical improvement in neuropathic pain conditions, although differences in response between trigeminal and postherpetic neuralgia should be interpreted cautiously given the limited number of trials and potential heterogeneity. Similarly, while higher doses demonstrated larger point estimates in certain analyses, substantial variability across studies precludes firm conclusions regarding dose-related biological effects. Overall, current mechanistic evidence supports the hypothesis that BTX-A exerts neuromodulatory actions at both peripheral nociceptor terminals and central pain processing pathways 5, 41. However, direct causal links between molecular mechanisms and clinical subgroup differences remain to be established.

The present findings may have implications for the management of patients with refractory neuralgia. Across the included trials, BTX-A was associated with reductions in pain intensity and frequency, as well as improvements in selected patient-reported outcomes. These observations suggest that BTX-A could be considered as a potential adjunctive or second-line option in individuals who experience inadequate response or tolerability issues with conventional oral therapies such as carbamazepine, gabapentin, or pregabalin 42. However, given the limited number of trials and modest sample sizes, clinical decision-making should remain individualized. The observed reduction in acute analgesic use may indicate a potential role for BTX-A in decreasing reliance on systemic medications. This consideration is relevant because long-term use of anticonvulsants or opioids is associated with adverse effects and safety concerns 43, 44. Nevertheless, the extent to which BTX-A can meaningfully reduce long-term pharmacotherapy burden requires confirmation in larger and longer-term studies. Although several trials reported sustained effects for up to approximately twelve weeks, follow-up beyond this period remains limited. Therefore, while periodic injection strategies may be feasible, the optimal treatment interval for neuralgia has not yet been established. Finally, improvements in sleep quality and quality of life observed in this analysis suggest that BTX-A may influence broader aspects of patient well-being; however, these multidimensional outcomes should be interpreted within the constraints of the available evidence.

Several important limitations should be acknowledged. First, the total number of eligible randomized controlled trials was limited, and many studies enrolled relatively small sample sizes. This restricts statistical power and increases the risk that pooled estimates may be influenced by small-study effects. Second, substantial heterogeneity was observed in several pooled and subgroup analyses, particularly in higher-dose categories and analgesic consumption outcomes. Variability in injection techniques, dose distribution, anatomical targeting, formulation differences, and baseline patient characteristics may have contributed to this inconsistency. Third, subgroup analyses were frequently based on a small number of trials, and in some instances on a single study. As such, these findings should be considered exploratory and hypothesis-generating rather than confirmatory. Fourth, outcome measurement was not fully standardized across studies. While most trials used validated pain scales, differences in assessment timing, diary methods, and reporting approaches may have introduced measurement variability. Fifth, follow-up duration was generally limited to approximately twelve weeks, and only a small number of studies extended observation beyond this period. Consequently, conclusions regarding long-term durability, optimal retreatment intervals, or cumulative dosing effects remain uncertain. Sixth, although all included trials were randomized, blinding procedures varied and may have been compromised in certain settings due to visible local effects of BTX-A, potentially introducing performance bias. Finally, most studies were conducted in single-center settings and within specific geographic regions, which may limit generalizability to broader and more diverse patient populations. These methodological and clinical constraints underscore the need for cautious interpretation of the pooled findings.

## Conclusion

This updated meta-analysis suggests that botulinum toxin type A is associated with improvements in pain intensity, pain frequency, sleep quality, and patient-reported outcomes in individuals with neuralgia. A reduction in acute analgesic use was also observed. However, the available evidence is based on a limited number of randomized controlled trials with relatively small sample sizes and short follow-up durations. Substantial heterogeneity was present in certain subgroup analyses. Therefore, larger multicenter trials with standardized injection protocols, longer follow-up periods, and independent replication are required to confirm these findings and better define the role of BTX-A in clinical practice.

## Figures and Tables

**Table 1:** Characteristics of included studies

Author (year) / Country/ Diagnosis	Inclusioncriteria	Exclusion criteria	Sample size(% of male)/ age	Studydesign	Placebo using	Concomitant medication	Intervention/ Duration	Main Results	Secondary Results
Xiao (2010) / China / PHN	With clinically confirmed PHN (persistent pain >3 months after rash healing), VAS pain score >5/10 for >3 months, and history of failed pharmacotherapy or intolerable side effects.	Contraindications to BTX-A (e.g., myasthenia gravis, neuromuscular disorders), hypersensitivity to BTX-A, coagulation disorders, local infection, major depressive or anxiety disorder, history of drug or alcohol abuse.	P: 20 (45) I: 20 (55) lidocaine: 20 (40) / P: 67 ± 12.1 I: 70 ± 15.4 lidocaine: 65 ± 14.2	RCT/Double-blind/ Placebo/ 3 arm trial	0.9% saline injections	No other neuropathic pain drugs were initiated or adjusted during the trial.	Subcutaneous BTX-A injections (5 U/mL; maximum dose 200 U) into affected dermatome. Follow-up lasted 3 months.	BTX-A significantly reduced VAS pain scores compared with lidocaine and placebo at day 7 and at 3 months. Pain relief began around days 3-5, peaked at day 7, and remained stable for 3 months.	1. Sleep time improved significantly in BTX-A compared with other groups at day 7 and 3 months.2. Opioid use was lowest in BTX-A group (21.1% at day 7; 19.4% at 3 months) compared with lidocaine (52.6% and 35.7%) and placebo (66.7% and 39.6%).3. No serious adverse events.
Wu (2012) / China / Classical TN	Adults with TN diagnosed by ICHD-2, pain intensity >4 on VAS, >4 attacks per day, failure of recent standard therapy (carbamazepine, gabapentin, or opioids).	Neuromuscular disorders, use of agents interfering with neuromuscular function, local infection or skin problems at injection site, pregnancy, nursing, planning pregnancy, unwilling to use contraception, or medical conditions increasing risk of BTX-A.	P: 20 (50) I: 22 (40.9) / P: 58 ± 16.91 I: 59.14 ± 12.58	RCT/Double-blind/ Placebo	0.9% saline injections	Concomitant meds allowed if stable (carbamazepine, gabapentin, opioids); no changes during trial.	BTX-A 75 U; single dose. Followed for 12 weeks.	1. VAS pain scores: significantly reduced from week 2 onward, sustained through week 12.2. Attack frequency: significantly reduced from week 1 onward, sustained through week 12.	1. Patient Global Impression of Change (PGIC): 77.3% in BTX-A group reported much/very much improved vs. 20% placebo2. Response rate (>50% reduction in pain score): 68.2% BTX-A vs. 15% placebo3. Adverse events: no serious safety concerns.
Apalla (2013) / Greece / PHN	Adults >18 yrs, PHN >3 months after rash healing, baseline VAS >7	Cranial nerve involvement, severe non-PHN pain, skin disorders affecting injections, pregnancy or lactation, recent treatments (<30 days), only paracetamol up to 1000 mg/day allowed during trial.	P: 15 (66.6) I: 15 (53.3) / P: 77.5 ± 8.2 I: 73.2 ± 10.5	RCT/Double-blind/ Placebo	0.9% saline injections	Concomitant treatments discontinued >30 days prior; only paracetamol <1000 mg/day allowed during study.	BTX-A 100 U, injected subcutaneously in affected area; single dose. Follow-upfor 20 weeks.	1. 87% of BTX-A group achieved >50% VAS reduction vs. 0% in placebo (p<0.001).2. Median time to 50% VAS reduction: 7.4 days.3. Pain reduction maintained ~16 weeks.	1. Sleep scores improved significantly from baseline to week 2, maintained up to 12 weeks.2. Well tolerated, only transient injection pain.3. No systemic adverse effects.
Shehata (2013) / Egypt / Intractable idiopathic TN	Idiopathic TN, trigger points elicited by mild tactile stimulation, intractable (failure to achieve >50% reduction in pain intensity or attack frequency despite proper drugs and dosages for 3 months)	Patients responsive to medical treatment, pregnancy, symptomatic TN, lack of coherence for follow-up.	P: 10 I: 10 / Mean age: 45.95 ± 10.02	RCT/Double-blind/ Placebo	0.9% saline injections	11 patients were treated with carbama-zepine (600-1400 mg), some in combination with gabapentin (400-1200 mg) and baclofen (30-75 mg), while nine received oxcarbazepine (900-1800 mg), occasionally combined with gabapentin.	BTX-A 100 U, subcutaneous injections; single dose. Follow-up 12 weeks.	1. VAS reduction: mean decrease 6.5 vs. 0.3 in placebo (*p*<0.0001).2. Paroxysm frequency reduction: significant from week 2, sustained through week 12.	1. Significant decrease in weekly acute medication use (*p*<0.0001).2. Significant improvement in QoL scale (*p*<0.0001).
Zúñiga (2013) / Argentina / ETN	Adults >18 yrs, clinical diagnosis of ETN, normal neurological exam, MRI to exclude secondary causes, refractory to usual treatments.	Symptomatic TN (secondary causes), responders to usual therapy, recent changes in medical regimen (<2 months), pregnancy, hypersensitivity to BTX, contraindication to invasive procedures.	P: 16 (62.5) I: 20 (45) / P: 66.06 ± 14.16 I: 64.5 ± 12.94	RCT/Double-blind/ Placebo	0.9% saline injections	Stable background pharmacotherapy maintained.	BTX-A 50 U, subcutaneous injections; single dose. Follow-up 12 weeks.	1. VAS scores: significant reduction by month 3 (*p*=0.01).2. Paroxysm frequency: reduced from 29.1/day to 7.1/day (*p*<0.001) vs. placebo nonsignificant (*p*=0.19).	1. Functional impact scale: improved significantly in BTX group (*p*<0.001) vs. placebo nonsignificant.2. Relapse: HR for relapse with BTX 0.35 (*p*=0.017).3. Adverse events: no serious events.
Zhang et al. (2014) / China / Classical TN	Adults >18 yrs, classical TN, disease course >4 months, mean VAS >4, >4 attacks/day, failure of recent standard treatments (carbamazepine, gabapentin, opioids), stable regimen at baseline.	Neuromuscular diseases (myasthenia gravis, motor neuron disease, Lambert-Eaton), infection/skin problems at injection site, drugs affecting neuromuscular junction within 7 days, unstable systemic disease, significant psychiatric disorder, substance abuse, pregnancy/lactation/planning pregnancy.	P: 27 (51.58) I (25U): 25 (40) I (75U): 28 (42.86) / P: 58.41 ± 11.74 I (25U): 58.16 ± 11.5 I (75U): 62.64 ± 13.3	RCT/Double-blind/ Placebo/ 3 arm trial	0.9% saline injections	Continued stable background meds (carbamazepine, gabapentin, opioids); no new drugs allowed during study.	BTX-A 25 U or 75 U, single dose. Followed for 8 weeks.	1. VAS pain scores reduced significantly in both BTX-A groups vs placebo as early as week 1, sustained to week 8.2. No significant difference between 25U and 75U groups.3. Response rate (>50% VAS reduction): 70.4% (25U), 86.2% (75U), vs 32.1% placebo.	Adverse events: no discontinuations.
Chen (2022) / China / PHN	PHN pain >1 month after rash healing, ID-Pain >2, age >18 years, VAS >4.	History/family history of neuromuscular junction disease, recent drugs affecting NMJ or aminoglycosides (<2 wks), coagulation disorders, prior BTX-A (<6 months), severe systemic disease (heart, liver, renal failure), pregnancy/lactation, comorbid pain conditions, allergy to BTX-A or common analgesics, severe psychiatric illness, alcohol/drug abuse.	P: 50 (52) I: 50 (52) / P: 72.28 ± 7.43 I: 72.20 ± 6.57	RCT/Single-blind/	pulsed radiofrequency (active control)	Both groups received conventional drug treatment as background therapy: mainly gabapentin, tramadol, or oxycodone. Patients could adjust analgesic doses as needed during follow-up.	BNT-A group: 100 U, subcutaneous injections; single dose.RF group: pulsed radiofrequency at corresponding spinal segment.	1. Both groups had significant VAS pain reduction at 1, 4, 12, and 24 weeks vs baseline (*p*<0.05). 2. No significant difference between groups at any time point (*p*>0.05)	1. Similar opioid/gabapentin use at 24 weeks.2. Adverse events: pneumothorax (RF, resolved in 1 week); transient limb weakness (BNT-A, resolved by 12 weeks).

BTX-A: Botulinum Toxin Type A; ETN: Essential Trigeminal Neuralgia; I: Intervention; ICHD: International Classification of Headache Disorders; ID-Pain: Identifying Pain Questionnaire; P: Placebo; QoL: Quality of Life; PHN: Postherpetic neuralgia; TN: Trigeminal Neuralgia; VAS: Visual Analog Scale;

## Data Availability

Data included in article/referenced in article.
